# Evaluation of anthropometric measurements and clinical tests in the diagnosis of difficult airway in patients undergoing head and neck surgery

**DOI:** 10.55730/1300-0144.5367

**Published:** 2022-01-25

**Authors:** Şükrü Mert BAŞPINAR, İlkben GÜNÜŞEN, Demet SERGİN, Asuman SARGIN, Sacittin Taner BALCIOĞLU

**Affiliations:** Department of Anesthesiology and Reanimation, Faculty of Medicine, Ege University, İzmir, Turkey

**Keywords:** General anesthesia, head and neck surgery, difficult airway, difficult intubation, difficult laryngoscopy

## Abstract

**Background/aim:**

The aim of this study is to research the incidence of difficult airways and the effectiveness of anthropometric measurements and clinical tests used to predict difficult airways in patients undergoing head and neck surgery.

**Materials and methods:**

This study was performed on a total of 200 patients over the age of 18 who underwent head and neck surgery between December 2019 and March 2020. The demographic data of the patients in the preoperative period, previous operations/radiotherapy history applied to the head and neck region, and obstructive sleep apnea syndrome symptoms were recorded. In the physical examination, the jaw structure, mouth opening, jaw movement, and tooth structure of the patients, modified Mallampati classification, head and neck movements, neck circumference, thyromental and sternomental distance, atlantooccipital joint mobility, upper lip bite test, Wilson risk scoring, and Cormack–Lehane classification were evaluated.

**Results:**

The difficult laryngoscopy rate was identified as 19%, and the difficult intubation rate as 8%. Operation history related to head and neck (p = 0.002), presence of at least two of the obstructive sleep apnea syndrome symptoms (p = 0.008), Modified Mallampati score (p = 0.009), Wilson risk score (p = 0.004), upper lip bite test (p < 0.0001) and mouth opening (p = 0.001) were found to be associated with difficult laryngoscopy. Modified Mallampati score (p = 0.002), Wilson risk score (p < 0.0001), upper lip bite test (p < 0.0001), mouth opening (p < 0.0001), sternomental distance (p = 0.003), Atlantooccipital joint mobility (p = 0.001), and Cormack–Lehane classification (p < 0.0001) were found to be associated with difficult intubation. According to multiple logistic regression analysis, the results obtained for sternomental distance and mouth opening were OR 0.8, 95% CI 0.6–1.1 and OR 0.2, 95% CI 0.1–0.4, respectively.

**Conclusion:**

In patients who underwent head and neck surgery, it was observed that the frequency of difficult airway was higher, and particularly the Modified Mallampati score, Wilson risk score, upper lip bite test, and mouth opening were associated with both difficult laryngoscopy and difficult intubation.

## 1. Introduction

The most common cause of anesthesia-related complications is airway management mistakes, which account for 30%–40% of deaths [1]. Although there is no standardized definition of difficult airway in the literature, the American Society of Anesthesiologists (ASA) defines difficult airway as difficult mask ventilation in a patient, difficulties in inserting supraglottic airway devices, difficult laryngoscopy, and/or difficult endotracheal intubation [2]. Although the frequency of difficult airway is reported to be between 1.5% and 13.5% in the general population, it may differ according to the clinical and physical characteristics of these patients.

It is stated that this rate is 10–20 times higher in people with risk factors. Among some clinical indicators and measurements used in the prediction of difficult airway in the preoperative period, which are considered as risk factors, it can be counted patients’ high Mallampati classification, wide neck circumference, high body mass index (BMI), aging, male gender, short thyromental and sternomental distance, missing teeth, protrusion of the mandible, limited mouth opening, obstructive sleep apnea syndrome, and Wilson score [3–6]. It has been reported that the incidence of difficult airway occurrence is higher in patients with these risk factors [2].

The most important concern for anesthesiologists in patients undergoing surgery is airway management. This is a much bigger problem particularly in surgeries related to the head and neck region. Since there is limited information about patients who underwent head and neck surgery in the literature, the aim of this study is to determine the incidence of difficult airway and risk factors for difficult airway in individuals who underwent head and neck surgery in the otolaryngology operating room.

## 2. Materials and methods

After the university ethics committee approval (dated 17.04.2019 and decision no: 19-4.1T/41), this study, which was conducted cross-sectional between December 2019 and March 2020, included 200 ASA I–III class, over the age of 18 patients who underwent head and neck surgery.

Patients with limited mouth opening (<1 cm) and planned awake fiberoptic intubation, rhinoplasty and middle ear operations were not included in the anesthesia examination during the preoperative period. Preoperative demographic data of the patients (age, gender, weight, height, BMI), previous operations applied to the head and neck region and radiotherapy history, patients with a definite diagnosis of obstructive sleep apnea syndrome (OSAS) and/or OSAS findings (snoring, apnea during sleep), daytime sleepiness, cognitive dysfunction, decrease in job performance, low quality of life) were recorded in the case report form as “possibility of OSAS”. In physical examination, patients’ jaw structure (prognathia, micrognathia, etc.), tooth structure (missing tooth, protruding tooth, etc.), Modified Mallampati classification (MMS) [7,8], Wilson risk scoring (weight, head and neck movement, mandible structure, tooth structure, jaw movement) was recorded [9]. Additionally, it is recorded as thyromental distance (TMD) [10], sternomental distance (SMD) [11], atlantooccipital joint mobility (AOJM) (no decrease (> 35^o^), 1/3 decrease (22–34^o^), 2/3 decrease (12–21^o^), no extension (<12^o^) [11], neck circumference, mouth opening and upper lip bite test (Class I: upper lip with lower incisors can be bitten above the vermillion line, Class II: upper lip with lower incisors can be bitten below the vermillion line, Class III: cannot bite the upper lip with lower incisors) [12].

Standard anesthesia induction was applied to the patients who were taken to the operation room after preoxygenation with 100% O_2_ for 2 min or 4 deep breaths. Considering the difficulty in mask ventilation, difficult laryngoscopy (as Cormack-Lehane 3 and 4), and difficult tracheal intubation, the patients were evaluated and recorded in the case report form. According to ASA guidelines [1,2], the difficulty experienced in ventilation as a result of gas leakage from the mask that cannot be prevented or excessive resistance at the gas inlet and outlet causes insufficient ventilation. Signs of insufficient face mask ventilation are listed as follows: absence or insufficient chest movement, absence of respiratory sounds, cyanosis, gastric air intake or dilatation, decreased or insufficient oxygen saturation (SpO_2_), inability to see ETCO_2_, hemodynamic changes accompanying hypercarbia or hypoxia (hypertension, tachycardia, arrhythmia). Based on these guidelines, we also defined difficult mask ventilation as the difficulty experienced in ventilation as a result of unavoidable gas leakage from the surface of the mask in contact with the face or excessive resistance at the gas inlet and outlet. Difficult laryngoscopy was defined as inability to visualize any part of the vocal cords after repeated multiple attempts in conventional laryngoscopy or Cormack-Lehane 3 and 4 [9]. In our study, we defined Cormack-Lehane 3 or 4 as difficult laryngoscopy. On the side of intubation, we categorized the difficult intubation as the case where an experienced anesthesiologist achieves intubation after at least 3 attempts [2]. All anthropometric measurements, clinical tests, airway applications, and evaluations were performed by two experienced anesthesiologists (ŞMB was performing the measurements and tests, and DS has performed mask ventilations and intubations), and they were recorded in case report form.

Statistical analysis was made by using IBM SPSS Statistics 25.0 (IBM SPSS Statistics for Windows, Version 25.0. Armonk, NY: IBM Corp.) package program. The significance level was determined as α = 0,05 in all analyses. Numerical data in the study were summarized by using mean and standard deviation, and categorical data by using frequency and percentage values. The assumption of normality of quantitative variables was examined separately in the easy-difficult laryngoscopy/intubation groups with the Shapiro-Wilk test, and the Mann–Whitney U test was used accordingly. The receiver operating characteristic (ROC) curve was used to evaluate variables’ ability for classifying laryngoscopy/or intubation status. Area under the curve (AUC) was summarized with 95% confidence intervals (CI). For statistically significant quantitative variables Youden index was used to determine the cut-off point that optimizes the variable’s differentiating ability by giving equal weight for sensitivity and specificity. Chi-square/Fisher exact test was done for comparison of categorical/nominal data between groups classified according to intubation/laryngoscopy difficulty. The effect size for the Chi-square results was calculated by Cohen’s $w=\sqrt{\chi^{2}/N}$ formula. According to this formula, it is classified as w = 0.10 small, w = 0.30 medium and w = 0.50 large effect size [13]. For difficult intubation and/or difficult laryngoscope, multiple logistic regressions were created with variables that were found to be clinically and statistically significant, and the results were given with odds ratios and 95% confidence intervals for these ratios. The “Linear-by-Linear association” test was used to evaluate whether the predictive rate of difficult intubation increases as the category value of the scores used to predict difficult intubation and/or difficult laryngoscopy. Clustered bar charts are used to visualize these associations. Lastly, for predicting difficult intubation and/or difficult laryngoscopy, these variables were converted to binary format according to the cut-offs suggested by the literature for quantitative variables and scores, sensitivity, specificity, positive, and negative predictive value of the binary outcomes, and accuracy values were calculated with MedCalc online calculator (https://www.medcalc.org/calc/diagnostic_test.php), and they are given with 95% confidence intervals.

## 3. Results

A total of 200 adult patients, who underwent elective head and neck surgery (mass excision, dissection, biopsy, etc.) under general anesthesia between 01.11.2019 and 30.09.2020, were included in the study. 131 (65.5%) of the patients were male, and 69 (34.5%) were female. The mean age of patients was found to be 55.42 ± 14.2 years, and their mean body weight was calculated as 77.07 ± 15.28 kg. Their mean height was 169.06 ± 9.2 cm, and their mean body mass index (BMI) was 26.97 ± 5.1 kg/m^2^. Demographic characteristics of patients were not statistically significant between easy-difficult laryngoscopy/intubation groups (Table 1).

While difficult mask ventilation was not observed in any of the patients, difficult laryngoscopy was detected in 38 patients (19%) and difficult intubation was detected in 16 patients (8%), and difficult laryngoscopy was also observed in all patients with difficult intubation. While 13 of 16 patients with difficult intubation were intubated using the sellick maneuver and stylet, 2 patients were intubated using a videolaryngoscope, and tracheotomy was performed in one patient.

According to the pathology and the surgical intervention area, the patients were examined in 3 groups: intraoral/pharynx/larynx region, neck region and face region. The number of patients who had an operation from the intraoral/pharynx/larynx region was 135 (67.5%), 47 (23.5%) patients from neck region and 18 (9%) patients from face region were operated. According to the applied surgical procedure, patients were divided into 3 groups as mass excision (benign/malignant), neck dissection with mass excision, and diagnostic biopsy. Mass excision applied 82 (41%) patients, neck dissection with mass excision applied 29 (14.5%) patients and diagnostic biopsy applied 89 (44.5%) patients.

When the patients were evaluated according to the previous operation and/or radiotherapy history of the head and neck region, it was determined that 45 (22.5%) of 200 patients had previously undergone an operation related to the head and neck, 3 patients (1.5%) had a radiotherapy history, 4 patients (2%) had both undergone radiotherapy and operation history.

The operation history was associated with the difficult laryngoscopy (p = 0.002), and it shows that having an operation history increases the probability of having a difficult laryngoscopy (Table 2).

Regarding OSAS, none of the 200 patients had a prior diagnosis of OSAS. However, when the OSAS findings [14] were examined, it was seen that 54.5% of the patients had at least 2 of the OSAS findings (the most common was snoring, daytime fatigue, and sleepiness) and these patients were evaluated as patients with the possibility of OSAS in the study (Table 2). In a similar way, patients with OSAS had a higher rate of difficult laryngoscopy than those without (p = 0.008; 11.0% vs. 25.7%).

**Figure 1:**
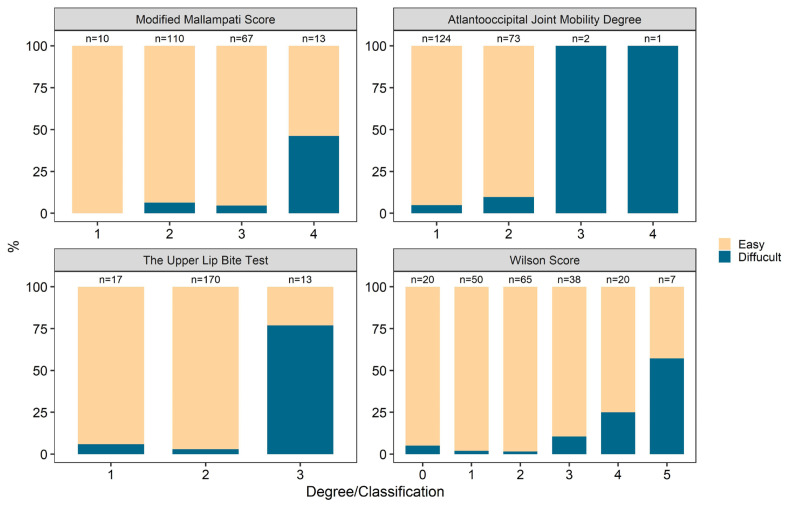
Distribution of Patients with Difficult Intubation according to Modified Mallampati Scoring, Atlantooccipital Joint Mobility, Wilson Score, and Upper Lip Biting Test.

There was no anatomical anomaly (presence of prognathia, micrognathia) in the jaw structure in any of the patients included in our study. Dental defect (missing and/or prominent teeth) was not associated both difficult laryngoscopy and difficult intubation (p = 0.34 and p = 0.81). When the patients were examined according to their MMS, there was a significant association between the MMS and difficult laryngoscopy/intubation (p = 0.009 and p = 0.002). It was found that the incidence of difficult laryngoscopy/intubation increased as the Mallampati score increased. Although linear by linear association tests were significant, discriminative ability of it was AUC = 0.62 for laryngoscopy and was AUC = 0.66 for intubation and these AUC values below the acceptable limits. There was no significant association between AOJM and difficult laryngoscopy, however, it was observed that the probability of difficult intubation increased as the degree increased in AOJM (p < 0.001). According to the Wilson scoring [9] 25, the highest score among the included patients was found to be 5, and the total number of patients with a Wilson score of <2 was found to be 70 (35%) (Table 3). In terms of difficult laryngoscopy, the risk score was found to be <2 in 10 (26.3%) of 38 patients, and it was observed that difficult laryngoscopy increased as the score increased (p = 0.004). For difficult intubation, the number of patients with Wilson risk score <2 was found to be 2 (12.5%), which was significant (p < 0.0001). The upper lip bite test (ULBT) was found to be significant in terms of both difficult laryngoscopy and difficult intubation (p < 0.0001) and it was determined that the probability of both increases as the classification increases (Table 3) (Figure 1). In our study, patients who were evaluated as 3 and 4 according to Cormack-Lehane classification were defined as difficult laryngoscopy, and 38 (19%) of 200 patients in total were accepted as difficult laryngoscopy. In terms of intubation, difficult intubation was observed in 14 (38.9%) of 36 patients with Cormack–Lehane 3, and in both 2 (100%) of 2 patients with Cormack-Lehane 4. It was found that as the Cormack–Lehane class increased, the difficult intubation rate increased as well (p < 0.0001) (Table 3).

The mean values for TMD, SMD, neck circumference, and mouth opening of 38 patients with difficult laryngoscopy and 16 patients with difficult intubation were shown in Table 4. Mouth opening was significant for difficult laryngoscopy and difficult intubation, and SMD, on the other side, was significant for difficult intubation. SMD and mouth opening were statistically significant for difficult intubation, and they were further analyzed by using multivariable logistic regression. According to multiple logistic regression analysis, the results obtained for sternomental distance and mouth opening were OR 0.8, 95% CI 0.6–1.1 and OR 0.2, 95% CI 0.1–0.4, respectively.

**Figure 2:**
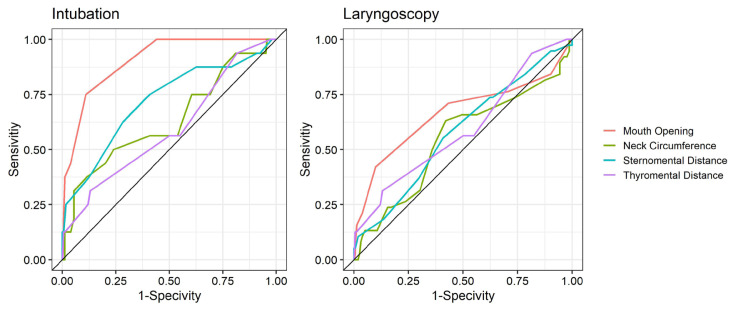
Physical examination measurements of patients and reference value showing with ROC curve.

The sensitivity, specificity, positive and negative predictive values of MMS, SMD, TMD, neck circumference, Cormack-Lehane classification, mouth opening, and Wilson Risk scoring in predicting difficult intubation or difficult laryngoscopy are shown in Table 5. It is seen that while among the tests applied for difficult laryngoscopy prediction the Wilson Risk Score (73.68%) had the highest sensitivity, TMD had the highest specificity (99.38%) and the highest positive predictive value (66.67%). Only the negative predictive value of Wilson Risk scoring was found above 90%. As a result of the ROC analysis, it was determined that the measurement of mouth opening was statistically high in predicting difficult laryngoscopy (AUC = 0.66). It was found that among the tests applied for difficult intubation prediction Cormack-Lehane classification (100%) had the highest sensitivity, TMD had the highest specificity (99.46%), and the highest positive predictive value (66.67%). Negative predictive values of all tests were found to be above 90%. As a result of the ROC analysis, it was found that SMD, Cormack–Lehane classification, Wilson Risk Scoring, and mouth opening were more likely to predict difficult intubation (Figure 2). AUC = 0.5 indicates insignificant, 0.6 ≥ AUC > 0.5 weak, 0.7 ≥ AUC > 0.6 acceptable, 0.8 ≥ AUC > 0.7 strong, AUC > 0.9 indicates perfect correlation. Accordingly, the SMD, Cormack–Lehane, Wilson Risk classification and AUC values of the mouth opening are 0.71, 0.94, 0.79, and 0.9, respectively (Table 5).

## 4. Discussion

In this study, conducted on patients who underwent head and neck surgery, previous head and neck surgery history, and at least two of the OSAS symptoms, although not diagnosed, were found to be associated only with difficult laryngoscopy. MMS, Wilson risk scoring, ULBT, and mouth opening were determined as tests that can be used to predict both difficult laryngoscopy and difficult intubation. In addition, SMD, AOJM, and Cormack–Lehane classification were found to be significant in difficult intubation.

In our study, while no patient had difficult mask ventilation, rate of difficult laryngoscopy was found to be 19%, and difficult intubation was 8%. Although these results are higher than the rates stated in the literature, different results have been reported in the literature regarding the difficult airway incidence, as well [15–17]. Whereas, Cattano et al. [15] reported the incidence of difficult intubation as 1.2–3.8% in adult patients, Iseli et al. [16] reported that the difficult airway rate was 7% and difficult intubation was 1.1% in their study of 2145 patients who underwent head and neck surgery. The difficult laryngoscopy rate was found to be 7.9% in the study of Wilson et al. [9] on 631 patients, and the difficult intubation rate was found to be 3.7% in the study of Tekgül et al. [17], which included 622 patients undergoing elective surgery. However, in most of the studies that are conducted, there are differences in methologies and definitions such as difficult airway and difficult intubation. For instance, in Iseli’s study [16], it was examined only whether patients had a difficult airway or not, the physical characteristics (anatomical structures) of the patients, clinical tests, and measurements used to predict difficult airway were not evaluated. In the literature, there is no homogeneity in terms of the types of surgery performed and the number of patients in studies on difficult airways, as well. We included only patients undergoing head and neck surgery in our study and used ASA guidelines for difficult airway definition. We think that the difference in rates is due to this. Once again, Langeron et al. [18] reported the incidence of difficult mask ventilation as 5%, which they defined as oxygen saturation below 92%, significant gas leak, significant absence of chest movement, the need for two-hand ventilation technique, or a change in people who perform ventilation. El Ganzouri et al. [19], on the other hand, reported the prevalence of difficult mask ventilation as 0.07%, which they defined as the inability to obtain sufficient chest motion to maintain a clinically acceptable capnogram waveform despite optimal head and neck position, and providing mask ventilation with the use of oral airway. Again, Cattano et al. [15] found that the difficult mask ventilation rate in adult patients was 0.01%–0.5%. In our study, we did not observe difficult mask ventilation in any of our patients. As Langeron et al. [18] stated, there are quite a few studies investigating difficult mask ventilation in the literature. Due to the differences in the definition of difficult mask ventilation, different rates are reported in the literature, and the rates can be found quite low in general [15,19,20].

Iseli et al. [16] found that 42 (27.6%) of 152 patients with difficult airway had a radiotherapy history and solely 8 (19%) of them had difficult intubation and the relationship was statistically insignificant. In our study, although the number of patients with a radiotherapy history was lower than Iseli et al [16], we obtained statistically similar results. We determined that there was a relationship between patients with only head and neck operation history and difficult laryngoscopy. Hiremath et al. [20], who investigated the relationship between difficult airway and OSAS, showed that there was a significant relationship between difficult laryngoscopy and OSAS in 30 obese patients. However, this study included patients who had a definite diagnosis by polysomnography. In another study by Leong et al. [21], OSAS was found to be a risk factor associated with difficult tracheal intubation. In fact, most of the patients who undergo surgery are unaware that they have OSAS. Diagnostic polysomnography can be applied to people suspected of questioning some symptoms of OSAS and this method is accepted as the “gold standard” in diagnosis. However, this method is very costly and requires time [20]. None of our patients had a prior diagnosis of OSAS. However, when some symptoms of OSAS were questioned, it was seen that people with at least two of them (most frequent snoring, sleepiness, daytime fatigue) showed a statistically significant relationship with difficult laryngoscopy.

El-Ganzouri et al. [19] evaluated MMS, mouth opening, TMD, and Cormack–Lehane scoring in terms of difficult airway in 10507 patients. Patients with a Cormack–Lehane score of 3 and 4 were defined as patients with airway difficulties. Among these parameters evaluated, mouth opening less than 4 cm, TMD less than 6 cm, and increase in MMS were found to be associated with difficult airway. In the study of Chhina et al. [22], which included 500 patients who underwent elective surgery, MMS in terms of difficult intubation, ULBT, TMD, SMD, neck circumference, and mouth opening were found to be associated with difficult intubation. Nevertheless, in a study by Savva [11] et al., it was stated that TMD was not related to difficult laryngoscopy, and SMD had higher specificity and sensitivity. In the study of Wilson et al. [9] involving 631 patients, the rate of difficult laryngoscopy was found to be 7.9% and it was stated that approximately 75% of the patients had a Wilson risk score of ≥ 2. In this study, a significant relationship was also found between mouth opening smaller than 4 cm and difficult laryngoscopy. On the other hand, Tekgül et al. [17] stated that there was no relationship between AOJM and BMI and difficult intubation in elective surgeries, however, along with TMD, as in our study, they reported that there was a statistically significant difference between MMS, ULBT, mouth opening, and SMD and difficult intubation. In a study conducted by Karkouti et al. [23] consisting of 461 patients, the difficult intubation rate was found to be 8.2%, which is similar to our study, and a statistical relationship was found with the mouth opening smaller than <4cm. Again, in the study of Rao et al. [24] involving 316 patients, it was stated that the rate of difficult laryngoscopy was 8.2%, while the MMS and mouth opening was found to be associated with difficult laryngoscopy, no difference was found between neck circumference and TMD. In another systematic review study by Detsky et al., it was shown that there is a strong relationship between ULBT and difficult intubation [25]. Similarly, in our study, these studies found that the increase in MMS, ULBT, Wilson risk score, and mouth opening <4 cm were found to be significant in predicting both difficult laryngoscopy and difficult intubation; however, a statistical relationship was not shown in terms of TMD and neck circumference, and it was determined that the SMD 12 cm is more significant in predicting difficult intubation. While Komatsu et al. [26] performed a small number (n: 64) of morbidly obese patients (BMI > 35) who underwent elective surgery, the rate of difficult laryngoscopy was found to be 31%, and they did not encounter difficult intubation in any of the patients. The mean neck circumference of the patients in the difficult laryngoscopy group was measured as 43.5 cm, the TMD as 8.5 cm, and the mouth opening as 4.6 cm, and no statistically significant correlation was found with difficult laryngoscopy. In our study, while the mean neck circumference of patients with difficult laryngoscopy was found to be 40.65 cm and the TMD 8.31 cm, unlike this study, our mouth opening measurements were less (mean 3.82 cm). Hence, we consider that it is associated with difficult laryngoscopy and difficult intubation. In our study, 32 patients (16%) with a mouth opening of <4cm were detected, and difficult intubation was observed in 12 (37%) of 32 patients, and difficult laryngoscopy was observed in 16 (50%). In another study conducted by Ezri et al. [27] consisting of 50 morbidly obese patients (BMI>35), it was stated that the rate of difficult laryngoscopy was 18%, TMD and mouth opening were insignificant; however, the neck circumference was significant. In this study, the mean neck circumference of the difficult laryngoscopy group was found to be 50 cm. Again, Brodsky et al. [28] supported the strong relationship between obesity and difficult intubation with neck circumference measurements. It has been stated that if the neck circumference is 40 cm, 5% difficult intubation, and if it approaches 60 cm, 35% difficult intubation is observed. In another study in the literature, neck circumference is not a statistically significant predictor for difficult laryngoscopy [29]. In our study and in Komatsu’s [26] study, the mean neck circumference of difficult laryngoscopy patients was lower than the mean determined by both Brodsky et al. [28] and Ezri et al. [27], and therefore, it may be considered to be insignificant. Hashim et al. [30] examined the AOJM in two groups as <35^o^ and >35^o^ in their study, and joint mobility was found to be < 35^o^ in 27 (54%) patients. However, difficult intubation was observed in only 5 (18%) of 27 patients and no statistically significant difference was found. In our study, AOJM was examined in 4 groups, and it was observed that the possibility of difficult intubation increased with increasing degree, and this relationship was significant. In the study of Hashim [30], we consider that the relationship may have been found to be insignificant since the definition of AOJM was defined as only 2 groups.

In a metaanalysis where Shiga et al. [5] compared 35 studies examining clinical tests and measurements used to predict difficult intubation, it was stated that the Wilson risk score could accurately predict easy intubation and laryngoscopy. In this metaanalysis, they accepted patients with Cormack–Lehane score 3 and 4 as difficult intubation in order to standardize the definition of difficult intubation. TMD, SMD, mouth opening, and Wilson risk scoring were examined, and it was found statistically significant in a total of 5 studies that as the Wilson risk score increased, the probability of difficult intubation increased. This relationship was shown with the ROC curve in the metaanalysis, and the AUC value was found to be 0.75. Also in our study, as the Wilson risk score increased, the probability of difficult intubation increased, and the AUC value was found to be 0.79. In this metaanalysis, TMD was examined in 17 studies, SMD and mouth opening, on the other hand, were examined in 3 studies. Even though the cut-off values for TMD vary between 4 cm and 7 cm, this value was determined as 6 cm in metaanalysis evaluation as in our study. When TMD was evaluated on the ROC curve, the AUC value was found to be 0.64, while the AUC value was 0.59 in our study. In the literature, AUC value of 0.6 ≥ AUC > 0.5 indicates the presence of a weak relationship, 0.7 ≥ AUC > 0.6 is acceptable, 0.8 ≥ AUC > 0.7 as a perfect relationship [31]. The reason why this relationship was not found stronger for TMD was explained as the presence of heterogeneity in measurement techniques and cut-off values. The AUC value of SMD was found to be 0.8, and it was determined as the test with the lowest negative predictive value (80%) among the parameters examined. As a conclusion, it has been reported that it is the best measurement that can be used to exclude difficult intubation. Also in our study, the AUC value for SMD was found to be 0.71, a high negative predictive value (93.33%) was obtained, and it was found significant in terms of difficult intubation. For mouth opening, on the other hand, the AUC value was found as 0.72 in the studies examined, and this value was 0.9 in our study. The reason for not finding a higher correlation in the meta-analysis was explained as the insufficient data on mouth opening [5].

In our study, we defined difficult laryngoscopy as Cormack–Lehane classes 3 and 4. When the literature is reviewed, it is seen that Cormack–Lehane 3 and 4 are accepted as difficult intubation in many studies. However, as stated in the study conducted by Wilson et al. [9], although there is a high correlation between difficult laryngoscopy and difficult intubation, difficult laryngoscopy does not always signify difficult intubation. In some patients, intubations were performed at a time without viewing the glottis. Even though we based on the definition of ASA as difficult intubation [1,2] in our study, we statistically determined that as the Cormack–Lehane class increased, the probability of difficult intubation increased, as well. Whereas difficult intubation was observed in all of our Cormack–Lehane class 4 patients, difficult intubation was not detected in 61% of 36 patients who were class 3, as Wilson et al [9] stated. It is found that the Cormack–Lehane classification has a positive predictive value of 42.11% and a negative predictive value of 100%. This shows us that Cormack–Lehane classes 1 and 2 can always indicate easy intubation; however, classes 3 and 4 may not always mean difficult intubation.

The most important limitation of this study is that it was conducted in a single center and only on the Turkish people. Different results can be obtained by adapting the findings of our study to multiple centers and human races. Another limitation of the study is the use of only ASA guidelines for difficult airway definition and other definitions in the literature are not included.

Consequently, it was observed that the incidence of the difficult airway was higher in patients who underwent head and neck surgery compared to the rates reported in the general population. While MMS, Wilson risk scoring, ULBT, and mouth opening were found to be significant tests that can be used to predict both difficult laryngoscopy and difficult intubation, SMD, AOJM, and Cormack–Lehane classification associated with difficult intubation, history of previous head-neck surgery, and having OSAS symptoms was found to be associated merely with difficult laryngoscopy.

## Figures and Tables

**Table 1:** Demographic data of the patients.

	Easy Laryngoscopy (n = 162)	Difficult Laryngoscopy (n = 38)	p	Easy Intubation (n = 184)	Difficult Intubation (n = 16)	p	Total Patients (n = 200)
**Age (years)**	55.84 ± 14.44	53.66 ± 12.97	0.42	55.43 ± 14.39	55.19 ± 12.18	0.94	55.42 ± 14.20
**Height (cm)**	169.01 ± 9.42	169.29 ± 8.33	0.52	169.30 ± 9.19	166.38 ± 9.15	0.18	169.06 ± 9.20
**Weight (kg)**	76.95 ± 14.96	77.58 ± 16.79	0.83	77.53 ± 15.09	71.75 ± 16.91	0.34	77.07 ± 15.28
**BMI (kg/m** **^2^** **)**	26.96 ± 5.08	26.99 ± 5.26	0.53	27.06 ± 5.05	25.86 ± 5.72	0.58	26.97 ± 5.11

All data presented with Mean ± Standard Deviation unless otherwise stated in the table, BMI: Body Mass Index.

**Table 2:** The comparison of operation and radiotherapy history, and OSAS between patients experienced difficult vs easy intubation/laryngoscopy.

	Easy Laryngoscopy (n = 162)	Difficult Laryngoscopy (n = 38)	p	Easy Intubation (n = 184)	Difficult Intubation (n = 16)	p	Total Patients (n = 200)
**Operation and Radiotherapy**			0.002			0.084	
No History	129 (79.6%)	19 (50%)		139 (75.5%)	9 (56.3%)		148 (74%)
Operation	28 (17.3%)	17 (44.7%)†		40 (21.7%)	5 (31.3%)		45 (22.5%)
Radiotherapy	2 (1.2%)	1 (2.6%)		2 (1.1%)	1 (6.3%)		3 (1.5%)
Operation and Radiotherapy	3 (1.9%)	1 (2.6%)		3 (1.6%)	1 (6.3%)		4 (2%)
**OSAS**			0.008			0.503	
Negative	81 (50%)	10 (26.3%)		85 (46.2%)	6 (37.5%)		91 (45.5%)
Positive	81 (50%)	28 (73.7%)*		99 (53.8%)	10 (62.5%)		109 (54.5%)

OSAS: Obstructive sleep apnea syndrome, column percentages were used

* p < 0.05,

† the observed frequency higher than expected according to z value of the adjusted residual of the cell (z > 1.96).

**Table 3:** Association between Easy-Difficult Laryngoscopy/Intubation and Patients’ Tooth Structure, Modified Mallampati Scoring, Atlantooccipital Joint Mobility, Wilson Score, Upper Lip Biting Test, Cormack–Lehane classification.

	Easy Laryngoscopy (n = 162)	Difficult Laryngoscopy (n = 38)	p	Easy Intubation (n = 184)	Difficult Intubation (n = 16)	p	Total Patients (n = 200)
**Tooth Structure**			0.34			0.81	
Normal	93 (57.4%)	25 (65.8%)		109(59.2%)	9 (56.3%)		118 (59%)
Defected	69 (42.6%)	13 (34.2%)		75 (40.8%)	7 (43.7%)		82 (41%)
**MMS**			0.009*			0.002*	
1	8 (4.9%)	2 (5.3%)		10 (5.4%)	-		10 (5%)
2	96 (59.3%)	14 (36.8%)		103 (56%)	7 (43.8%)		110 (55%)
3	51 (31.5%)	16 (42.1%)		64 (34.8%)	3 (18.7%)		67 (33.5%)
4	7 (4.3%)	6 (15.8%)		7 (3.8%)	6 (37.5%)		13 (6.5%)
**AOJM**			0.13			0.001*	
Degree- 1	102 (63%)	22 (57.9%)		118 (64.1%)	6 (37.5%)		124 (62%)
Degree- 2	60 (37%)	13 (34.2%)		66 (35.9%)	7 (43.8%)		73 (36.5%)
Degree- 3	-	2 (5.3%)		-	2 (12.5%)		2 (1%)
Degree- 4	-	1 (2.6%)		-	1 (6.2%)		1 (0.5%)
**Wilson Score**			0.004*			<0.0001*	
0	17 (10.5%)	3 (7.9%)		19 (10.3%)	1 (6.2%)		20 (10%)
1	43 (26.5%)	7 (18.4%)		49 (26.6%)	1 (6.2%)		50 (25%)
2	57 (35.2%)	8 (21%)		64 (34.8%)	1 (6.2%)		65 (32.5%)
3	28 (17.3%)	10 (26.3%)		34 (18.5%)	4 (25%)		38 (19%)
4	15 (9.3%)	5 (13.2%)		15 (8.2%)	5 (31.3%)		20 (10%)
5	2 (1.2%)	5 (13.2%)		3 (1.6%)	4 (25%)		7 (3.5%)
**ULBT**			<0.0001*			<0.0001*	
Class- 1	15 (9.3%)	2 (5.3%)		16 (8.7%)	1 (6.2%)		17 (8.5%)
Class-2	145 (89.5%)	25 (65.8%)		165 (89.7%)	5 (31.3%)		170 (85%)
Class-3	2 (1.2%)	11 (28.9%)		3 (1.6%)	10 (62.5%)		13 (6.5%)
**Cormack-Lehane**			-			<0.0001*	
**1**	80 (49.4%)	-		80 (43.5%)	-		80 (40%)
**2**	82 (50.6%)	-		82 (44.6%)	-		82 (41%)
**3**	-	36 (94.7%)		22 (11.9%)	14 (87.5%)		36 (18%)
**4**	-	2 (5.3%)		-	2 (12.5%)		2 (1%)

MMS: Modified Mallampati Scoring, AOJM: Atlantooccipital Joint Mobility, ULBT: Upper Lip Biting Test, Column percentages were used,

* p < 0.05.

**Table 4:** Neck circumference, mouth opening, thyromental and sternomental distance.

	Easy Laryngoscopy (n = 162)	Difficult Laryngoscopy (n = 38)	p	Easy Intubation (n = 184)	Difficult Intubation (n = 16)	p	Total patiens (n = 200)
**Neck Circumference (cm)**	40.04 ± 4.44	40.65 ± 5.36	0.36	40.34 ± 4.53	38 ± 5.24	0.074	40.16 ± 4.62
**Mouth Opening (cm)**	4.40 ± 0.69	3.82 ± 1.22	0.001*	4.41 ± 0.71	2.9 ± 1.05	<0.0001*	4.29 ± 0.84
**Thyromental Distance (cm)**	8.54 ± 1.02	8.31 ± 1.27	0.29	8.55 ± 1.03	7.96 ± 1.41	0.19	8.5 ± 1.07
**Sternomental Distance (cm)**	15.90 ± 2.12	15.18 ± 2.58	0.12	15.92 ± 2.09	13.93 ± 2.9	0.003*	15.76 ± 2.23

All data presented with Mean ± Standard Deviation in table,

* p < 0.05.

**Table 5:** Sensitivity, specificity, positive and negative prediction, AUC values of anthropometric measurements and clinical scorings.

Difficult Laryngoscopy	Se	Sp	PPV	NPV	AUC	p
Modified Mallampati Scoring	57.89%	57.14%	22%	86.67%	0.62	0.009*
Wilson Risk Scoring	73.68%	74.07%	40%	92.31%	0.63	0.004*
Sternomental Distance	7.89%	98.77%	60%	82.05%	0.57	0.12
Thyromental Distance	5.26%	99.38%	66.67%	81.73%	0.55	0.29
Neck Circumference	63.16%	48.15%	22.22%	84.78%	0.45	0.36
Mouth Opening	42.11%	90.12%	50%	86.9%	0.66	0.001*
**Difficult Intubation**						**p**
Modified Mallampati Scoring	56.25%	61.41%	11.25%	94.17%	0.66	0.002**
Cormack-Lehane Classification	100%	88.04%	42.11%	100%	0.94	<0.0001**
Wilson Risk Scoring	87.5%	69.57%	20%	98.46%	0.79	<0.0001**
Sternomental Distance	18.75%	98.91%	60%	93.33%	0.71	0.003**
Thyromental Distance	12.5%	99.46%	66.67%	92.89%	0.59	0.19
Neck Circumference	56.25%	46.20%	8.33%	92.39%	0.63	0.073
Mouth Opening	75%	89.13%	37.5%	97.62%	0.90	<0.0001**

Se: Sensitivity, Sp: Specificity, PPV: Positive Predictive Value, NPV: Negative Prediction Value, AUC: Area Under the Curve,

* p: p value for Difficult Laryngoscopy,

** p: p value for difficult intubation.
